# Supplementary material for: Are carbon emissions trading and green financial instruments synergistic? -Comprehensive quantitative research based on content analysis
Source: PLoS One. 2024 Mar 7;19(3):e0298601. doi: 10.1371/journal.pone.0298601 (PMC10919601; doi:10.1371/journal.pone.0298601)
Supplement: S1 Data — (ZIP) [file pone.0298601.s001.zip › Data/地级市Z_F工作报告.zip!/╒π╜¡/╠¿╓▌╩╨/└┤╫╘╣1⁄2╓┌║┼í╛┬φ┐╦╩2╛▌═°í┐_2015.docx]

各位代表：

　　现在，我代表台州市人民政府，向大会报告政府工作，请予审议，并请市政协委员和其他列席人员提出意见。

　　一、2014年工作回顾

　　过去一年，面对经济放缓、区域竞争加剧的压力，面对深化改革、加快转型升级的重任，市政府积极贯彻党的十八届三中、四中全会精神，全面落实市党代会决策部署，认真执行市人大各项决议，坚持稳中求进、改革创新、实干争先，突出抓改革、促转型、治环境、惠民生，经济社会发展取得新成绩。全市实现生产总值3387.51亿元，增长7.5%；地方一般预算收入265.21亿元，增长7.1%；城镇、农村常住居民人均可支配收入分别为39763元、19362元，增长9%、10.5%；单位生产总值能耗下降、主要污染物减排指标完成。

　　一年来，我们主要做了五方面工作：

　　（一）抓改革扩投资，推进更有动力的发展。

　　深化改革加快推进。小微企业金融服务改革创新走在全国前列，信保基金和信用信息共享平台运行，市金融投资公司、铁路建设投资公司组建。民间投资改革创新积极申报国家示范区。行政审批制度改革走在全省前列，“四张清单一张网”改革积极开展，市级行政审批事项从584项减少到216项，部门行政权力从8713项减少到4702项，非行政许可审批事项全面取消，网上政务大厅基本建成。商事登记制度改革成效显著，市场主体呈井喷式增长，新增7.2万户，公司制企业增长116%。政府机构改革加快推进，合并组建市市场监管局，台州经济开发区和绿心生态区、市基投公司和社投公司机构职责得到整合。市区交警和公安指挥一体化启动实施。公共资源配置市场化改革有序推进，财政专项资金竞争性分配改革开展试点，首宗海域使用权招拍挂项目完成出让。市属国有企业薪酬和用工管理得到规范。户籍制度改革、小产权房清理在玉环试点。农村综合改革深入推进，2753个村完成经济合作社股份合作制改革，市县两级农村产权交易所成立。黄岩列入省农村产权制度改革、促进土地集约利用示范试点，仙居列入省构建“三位一体”农合组织体系试点。开放合作不断加强。外贸出口增长3.4%，电子口岸启动建设。路桥成为国家出口机动车质量安全示范区，临海休闲用品产业基地成为国家外贸转型升级专业型示范基地。进出口企业诚信体系建设经验在全国推广。“世界500强及跨国公司台州行”举办。“浙商回归”成效显著，异地商会作用进一步发挥，引进省外到位资金200.57亿元，增长32.2%。重大项目不断突破。固定资产投资增长17.1%，居全省第二。列入省“411”重大项目计划数居全省第一，省重点建设新增补项目数占全省19%。头门港区开港，跨海大桥及首个3万吨级码头建成。大麦屿港区对台湾货运直航开通，玉环经济开发区综合物流园区保税仓库投入运营。椒江二桥、沿海大通道、104国道改建天台段全面通车，沿海高速台州段开工，内环路建设快速推进，金台铁路及头门港支线可研报告通过评审，市域铁路纳入浙江都市圈城际铁路规划，杭绍台高速工可获批。台州机场引进东航、国航飞机过夜站。三门核电一期、台州第二电厂一期、仙居抽水蓄能电站加快建设。洪家场浦、栅岭汪排涝调蓄工程积极推进。方溪水库、盂溪水库大坝主体工程开工。担屿涂围垦主体工程、洋市涂围垦海堤填筑基本完成。吉利V汽车、巨科铝轮毂项目加快建设，广汽吉奥、海正海诺尔项目开工，北车台州配套产业园项目签约，台州新机场、东海油气登陆、炼化一体化项目前期努力推进。发展平台加快构建。台州湾集聚区东部新区15平方公里大平台形成，二期15平方公里吹填工程开工，商业服务中心结顶，中小企业创业园加快推进。要素保障得到加强。新增表内外贷款882.15亿元，直接融资142.6亿元。新增建设用地3.65万亩，供应土地2.94万亩，消化批而未供土地1.58万亩，“三改一拆”拆后土地用于建设用地4121亩。

　　（二）抓创新促转型，推进更有质量的发展。

　　创新转型步伐加快。企业自觉转型意识提高，政府引领转型力度加大，环境倒逼转型成效明显。新增国家高新技术企业68家、国家企业技术中心2家、省级企业研究院17家、省级高新技术企业研发中心31家。微软（温岭）IT学院成立，与东南大学、中国工程物理研究院国家水处理中心共建台州技术转移中心协议签订，三门与中关村天合科技成果转化促进中心达成共建协议。市科技人才投资发展基金设立。专利授权量达到1.6万件。“工业强市”扎实推进。医药产业规划完成编制。“四换三名”深入实施，列入省“机器换人”重点项目数居全省第三，入选省“三名工程”试点企业数居全省第三。温岭列入国家机床工具产业集群区域品牌建设试点、“浙江制造”县域培育试点，鞋业整治提升成效明显。临海列入省综合性“腾笼换鸟”试点。椒江、临海、仙居列入省资源要素市场化配置综合改革试点。“两化融合”扎实推进，入选工信部试点企业数居全省第四。入围省优秀工业设计企业数居全省第一。新增驰名商标3件、著名商标31件、省名牌产品30个。对接资本市场活跃，新增上市企业3家、新三板挂牌4家。“个转企”6369家，“小升规”405家。全国质量强市示范城市创建通过验收。企业制（修）订国家标准9个、行业标准25个。建筑业总产值突破2000亿元，达到“建筑强市”标准。现代服务业加速发展。省市服务业集聚区加快建设。小微企业金融服务专营机构新设32家，村镇银行实现县域全覆盖，农村普惠金融、民间融资管理试点稳步推进，金融机构贷款余额增长13.1%，居全省第二。金融生态环境良好，不良率1.19%，远低于全省平均水平。

　　限额以上社会消费品零售总额增长14.5%，居全省第二，网络零售额增长80.3%。成功争创国家电子商务示范城市，电子商务产业园加快建设，天台成为省级电子商务示范县。快递业务量增长81%。温岭国际物流中心基本建成。天台山、神仙居创国家5A级景区列入预备名单，方山、蛇蟠岛成为国家4A级景区，神仙居国家旅游标准化试点通过验收。创建省级放心农贸市场22家。现代农业稳步发展。农业增加值增长2.1%，居全省第三。粮食安全责任制有效落实，早稻主产区面积增幅居全省第一。市粮食批发市场、中心粮库、农副产品集配中心努力推进。“两区”建设不断深化，建成高标准基本农田25万亩，新建粮食生产功能区8.82万亩、省现代农业综合区3个、主导产业示范区3个，建成特色农业“生态化产业化基地化”示范村51个、精品园10个。新增股份合作农场30家、家庭农场582家。与省农科院合作19个项目启动。“肥药双控”进展良好。农作制度创新重点乡镇和示范园区建设加快推进。农民学院成立。东海渔场修复暨“一打三整治”扎实开展，涉渔“三无”船舶取缔任务超额完成。建成标准渔港9座，渔港防灾减灾体系基本形成。

　　（三）抓整治优环境，推进更可持续的发展。

　　专项工作走在前列。“五水共治”获省首批“大禹鼎”，在全省率先完成垃圾河清理，清理垃圾河2600条4714公里，治理黑臭河168条284.2公里，新建污水收集管网446.7公里，日污水处理量新增4.83万吨，交接断面水质达标率上升到83.3%。天台成为省首批“清三河”达标县。“三改一拆”深入推进，拆违3514万平米，连续两年居全省第一，“三改”1621万平米，73个乡镇街道、2844个村达到“无违建”标准。交通治堵名列全省前茅，新增公共停车位7864个，市区公交一体化深入推进，新建公交站点164个，新增公交车辆100辆，公交分担率提高3.1个百分点。“多城同创”取得突破，国家卫生城市创建通过评估，国家环保模范城市创建通过技术评估，全省文明程度指数测评居同类城市第一。环境质量显著提升。主城区化工恶臭整治成果不断巩固，江口医化园区实施告别恶臭倒计时行动，椒江和临海医化基地循环化改造、仙居城南医药企业搬迁取得实质性进展，金属资源再生产业基地国家“城市矿产”示范试点、三门橡胶高新产业园建设加快。淘汰黄标车3.99万辆，完成有机废气治理工程78项、减排项目建设284个、重污染企业整治272家，整改、停产、取缔、关闭涉污企业1834家。空气质量优良率87.4%，5个月份进入全国74个监测城市前十名。生态市建设扎实推进。省森林城市创建通过验收。“四边三化”、“双清”行动成效明显。永宁江两岸绿带建成。临海成为首批省美丽县城试点，天台列入国家生态文明建设示范区，仙居成为首批国家公园试点。首次地理国情普查扎实开展，数字台州地理空间框架建成。中心城市加快建设。中央商务区3幢金融大楼建成，天盛、刚泰、腾达中心动工。商贸核心区银泰城市综合体、绿心生态区植物（雕塑）园和飞龙湖建设进展顺利。江北商务区、委羽山新区、高铁新区加快推进。中央山公园二期开园，星光耀广场综合体开工。台州湾集聚区月湖绿岛新区吹填工程加快。万达广场主体工程基本完工，麦德龙仓储式超市营业。美丽乡村建设深入开展。“清洁家园”活动深化，环卫保洁机制不断健全，垃圾分类减量无害化试点有序推进，农村生活污水治理居全省前列。创建美丽乡村精品村23个，建成美丽宜居示范村25个，农家乐乡村旅游成为新亮点。扶贫开发示范村和美丽库区建设成效明显。长潭水库连续43个月保持国家I类水质标准，库区38米线以下移民动迁安置完成。

　　（四）抓实事惠民生，推进更有福利的发展。

　　坚持为民办实事，十方面24个项目全面完成。社会保障不断完善。以创业带动就业，新增就业人数9.42万，城镇登记失业率2.21%。新农合与城镇居民基本医疗保险、被征地农民基本生活保障与职工养老保险、老农保与城乡居民养老保险实现并轨，大病医疗保险制度全面建立，全民参保登记行动启动。住房公积金提取、使用渠道不断拓宽。建成健康养老机构3家、城乡社区居家养老服务照料中心1574个。9家残疾人托养服务中心启动建设。社会事业全面进步。阳光招生常态化，建成学前教育合格乡镇21个，新增省义务教育标准化学校132所，义务教育均衡发展高标准推进。市文华小学投用，台州市直高中、台州电大新校园项目开工。公立医院改革和基层卫生综合改革平稳推进，分级诊疗试点扎实开展，社会办医势头良好，新增民营医疗机构25家、床位2229张，恩泽医院建成运行，市立医院与复星集团合作项目签约，市公共卫生中心、市妇女儿童医院（市妇幼保健院）开工。食品药品安全基层责任网络建设不断加强，食品安全检测资源整合、生鲜食品“放心柜”工程、百万学生饮食放心工程稳步推进。农副产品平价商店启动建设。“魅力台州”形象广泛宣传。文化产业加快发展，市电影发行放映公司、市人民影剧院改制基本完成，广播电视有线网络“一省一网”整合有序推进。文化惠民工程深入实施，农村文化礼堂建成226家。玉环被评为全国文化先进县。仙居成为“中国孝文化研究中心”实践基地。济公故里成为国家海峡两岸交流基地，大陈岛成为省级对台湾交流基地。体育产业积极培育，市少体校建设顺利推进。参赛省运会取得好成绩。浙江佛学院天台宗学院和浙江道教学院揭牌。统计工作得到加强，第三次经济普查完成。单独两孩政策稳妥实施，人口自然增长率5.09％。。外事侨务、对台事务、档案、气象、地方志工作取得新成绩，妇女儿童、老龄、老干部、关心下一代、慈善工作不断加强。平安建设深入开展。“三严三治三强”扎实推进，反恐防爆和应急处突工作得到加强，严厉打击污染环境、网络诈骗等突出犯罪，打霸护航、打防侵财、百城禁毒会战和反走私、反传销专项行动成效明显，刑事案发数明显下降。普法工作取得新成效，人民调解组织发挥积极作用，社区矫正规范化建设工作在全国推广。信访工作得到加强。安全生产“九打九治”行动扎实开展，三项控制指标全面下降。企业资金链风险有效防范和化解。防汛防台公共规则认真实施，社会化防台减灾成效显现。国防、海防、人防和民兵预备役工作不断加强。

　　（五）抓作风强服务，推进更有效能的发展。

　　切实改进政府作风。中央“八项规定”严格执行，党的群众路线教育实践活动认真开展，“四风”问题坚决纠正，24个专项整治和百项整改取得实效，一批群众反映突出的问题得到解决。因公出国、公务接待、公车管理等规定严格落实，“三公”经费下降32.2%。依法行政得到加强。认真办理人大代表建议、政协提案，办结率100%。决策咨询和法律顾问作用有效发挥，规范性文件制定公众参与度提高，政府合同合法性审查和行政复议规范化不断加强。政府服务有效改善。投资项目全程代办制度全面落实，公共服务窗口办事无休日制度全面推行。“五帮一化”服务企业、强基惠民村村帮制度建立。12345便民服务平台受理来电40万件，办理满意率92%。党风廉政建设责任制和廉政准则认真落实，对关键岗位开展电视问政等跟踪督评，行政审批、招投标、政府采购、公共资源交易监管加强，涉农资金、土地出让等强化审计监督。

　　各位代表！一年来，我们高度关注经济运行，加强研判，出台措施，落实责任，以坚定信心和担当顶住了经济下行的巨大压力，以坚持实干和拼劲赢得了来之不易的发展态势。我们体会最深的是：越是结构调整阵痛显现，越要保持定力，精准发力，持之以恒打好转型升级的组合拳，牢牢把握工作主动权；越要聚焦精力抓项目，集中资源办大事，锲而不舍下好科学发展的先手棋，积蓄台州发展新能量；越要顺应群众呼声，体现群众意愿，坚持不懈办好惠民利民的贴心事，提高人民群众满意度。

　　各位代表！2014年是台州撤地设市20周年。20年发展为台州未来跨越奠定了坚实基础。今天的台州，发展成就令人鼓舞，发展态势催人奋进，发展前景让人憧憬！台州发展的每一个变化，都得益于省委、省政府和市委的坚强领导，得益于市人大、政协的监督支持和全市上下的共同努力。在此，我代表市政府，向全市人民，向人大代表、政协委员，向各民主党派、工商联、各人民团体和社会各界人士，向驻台部队官兵、在台省部属各单位，向关心和支持台州发展的海内外朋友，表示衷心的感谢！

　　我们也清醒地看到，台州已经比较明显地进入了新常态，发展中还存在不少困难和问题。经济下行压力依然较大，稳中向好基础不够稳固，生产总值、地方财政收入、外贸进出口指标未能达到预期目标；深化改革任务繁重，创新动力不足，转型升级任重道远；中心城市辐射带动能力不强，城市基础设施仍不完善，提升承载能力任务艰巨；社会发展仍然滞后，关系群众切身利益的环境保护、食品安全、教育卫生等方面还存在不少薄弱环节，农民持续增收压力较大；法治政府建设需要加快，改进工作作风、提高行政效能、加强廉政建设还需付出更大努力。我们一定高度重视，采取有力措施加以解决。

　　二、2015年目标任务

　　2015年是全面深化改革的关键之年，是全面推进依法治国的开局之年，也是“十二五”规划的收官之年。市四届四次党代会明确了“一都三城”的战略目标，这是市委对台州未来发展的重大谋划，是对接国家“一带一路”和长江经济带战略的重要举措，是顺应发展趋势加快转型升级的迫切需要，是回应人民期待的现实要求。面对城市发展新定位，我们要凝聚共识、深化举措、加快推进。“一都三城”：“一都”是发展定位。现代化港湾都市区，立足台州三个海湾的自然禀赋，顺应湾区经济、都市区发展趋势，体现台州在省域城市格局中的重要地位和层级。“三城”是战略支撑。国际智造名城，立足台州扎实的制造业基础，把握世界新科技革命大趋势，体现国际化、智能化导向；海上丝路港城，立足台州丰富的港口岸线资源，对接国家战略，体现开放型、外向型导向；山海宜居美城，立足台州山海兼利的生态特色，落实“两美”浙江建设，体现可持续性、人文性导向。面对经济发展新常态，我们要全面把握新常态下速度变化、结构优化、动力转换的本质特征，坚持以更高质量、更好效益、更稳发展适应速度变化，着力打造更有效率的政务生态系统、更有活力的创业创新生态系统、更有魅力的自然生态系统、更加和谐的社会生态系统。面对依法治国新要求，我们要把法治作为核心价值追求，把依法办事作为基本要求，用法治思维和法治方式破解难题，推动政府依法行政、市民依法维权、社会依法治理，形成办事依法、遇事找法、解决问题用法、化解矛盾靠法的法治环境。只要我们谋长远之策，强发展之基，行惠民之举，就一定能够再创发展新业绩。

　　今年政府工作总体要求：全面贯彻党的十八大、十八届三中、四中全会和习近平总书记系列重要讲话精神，坚持以“八八战略”为总纲，认真落实市四届四次党代会决策部署，主动适应经济发展新常态，积极融入国家战略新布局，围绕“一都三城”的战略目标，坚持稳增长、调结构、抓改革、治环境、惠民生、强法治，着力实施十大行动，努力完成“十二五”规划目标，为“十三五”发展打下坚实基础。

　　经济社会发展主要预期目标：地区生产总值增长7.5%左右，地方一般预算收入增长7.5%左右，城镇、农村常住居民人均可支配收入分别增长8%以上、9%以上，快于经济增长，居民消费价格指数与省目标衔接，单位生产总值能耗下降、主要污染物减排完成省定目标。

　　重点实施十大行动：

　　（一）实施都市区建设行动。

　　推进都市区建设。深化城市规划研究，编制都市区建设实施方案，开展城市总体规划修改，完善土地利用总体规划，逐步形成“多规合一”规划体系。加快中心城市建设，推动市区融合发展，促进公共设施合理配置、共建共享，完善城市功能，提升都市生活品质。实施城乡建设“七五”工程，着力打造综合服务区、商贸核心区、生活休闲区三位一体联动发展的“都市核心”。推进市区重点区块建设。加快中央商务区建设，中央生态公园竣工。加快西商务区玉兰广场建设。商贸核心区银泰城市综合体结顶，启动心海生态文化园项目。推进环白云山和星明工业小区改造。加快绿心生态区植物（雕塑）园建设。推进高铁新区、江北中心商务区、江口区块和新前城市新区建设。加快西市场园区建设，加速飞龙湖成形。加快月湖绿岛新区建设。深化椒江两岸规划研究，推进江滨公园西进和滨江公园东延，加快椒北开发。加强市区西部山区保护开发。推进副中心城市和美丽县城建设，加快临海美丽县城示范城市建设，推动温岭现代化中等城市建设，支持玉环撤县设市，打造天台“名山福地”生态城，建设仙居中国山水画城市，加快三门“三港三城”建设。实施新一轮小城市试点三年行动计划，加强中心镇建设，积极培育一批省、市特色小镇。

　　完善城市基础设施。加快沿海高速台州段建设，金台铁路及头门港支线、杭绍台高速、台金高速东延二期开工，争取台金高速市区连接线、市域铁路开工，加快市区现代有轨电车项目前期。力争签订机场迁建协议。大力实施“一绕三环三纵三横”市区骨干路网规划，加快构建“快速路+主干路”的城市路网。完成市府大道东延至九塘坝，推进内环路、聚海大道、甲南大道、现代大道、桐江大道、海城路建设。完善城市生活设施配套，加快椒江污水处理厂三期、黄岩江口污水处理厂二期、市区天然气高压输配管网工程建设，推进城市生活垃圾焚烧扩建工程、市区餐厨垃圾处置中心、病死动物无害化处理设施建设。开展“海绵城市”建设。加强城市地下管线普查建档、规划建设和综合管理，推动地下空间开发利用。

　　提升城市管理水平。深入实施“多城同创”，构建常态化管理机制，巩固国家卫生城市、环保模范城市创建成果，加强市民素质教育，争创全国文明城市。深化“三改一拆”工作，实施“创无违建县三年行动计划”。扎实开展交通治堵，积极创建省级公交优先示范城市。加强城市智能化、精细化、人性化管理。

　　（二）实施创新驱动产业升级行动。

　　强化自主创新。创建省级创新型城市。大力培育高新技术企业、科技型中小企业，加强企业研发机构建设，新增国家高新技术企业30家、省级高新技术企业研发中心10家。发展网上技术市场，建设科技成果转化服务平台，推动科技服务业发展。引进大院名校共建创新载体，发挥浙大台州研究院、中科院台州应用技术研发与产业化中心等创新平台效用，深化与中国药科大学、东南大学战略合作，共建北大-台州金属燃料电池研究中心。积极谋划台州科技城，创建省医药高新区，推进台州经济开发区争创国家高新技术产业园区。加快培育创投基金，促进科技金融合作，与浙江清华长三角研究院建立科技金融创新众筹中心。全社会研发经费增长10%。推进“500精英计划”，加大高层次创新人才、团队引进和青年人才培养力度，扩大创业创新人才规模。

　　建设工业强市。坚持工业化和信息化、制造业与服务业深度融合，用智造升级“台州制造”，推动台州由工业大市向工业强市跨越。健全工业企业综合效益评价机制。抓好医化、汽摩等行业转型提升，推进36个县域块状经济向现代产业集群转型升级，变低小散为高精专，推动传统产业向中高端迈进。培育发展高铁、石化产业。依托32家上市公司，扶持培育行业龙头企业和“单打冠军”，推动并购重组，加快形成一批代表现代工业水准、主业突出、核心竞争力强的大企业、大集团。推进个转企、小升规、规改股、股上市，新增规上企业200家以上，帮助和推动有条件的企业加快上市步伐。推进“四换三名”，深化质量强市示范城市建设，加快国家电机及机械零部件、泵业检测中心建设。推动建筑业转型发展。

　　发展新兴产业。大力发展信息、环保、健康、旅游、时尚、金融、高端装备七大产业。加快发展信息经济，培育云计算、大数据、物联网等新兴业态，落实智慧城市发展规划，加强智慧水务、智慧管网建设。推进国家电子商务示范城市建设，打造电商全产业链，加快电商园区崛起，与清华大学共建电子商务交易技术国家重点实验室台州分支机构，力争成功申请互联网第三方支付牌照。加快电子口岸建设，争创国家跨境贸易电子商务试点城市。加快发展创意经济，鼓励工业设计、动漫影视文化创意产业发展。加快发展健康经济，推进健康产业发展。大力发展旅游业，按照打造国际旅游目的地和千亿产业的要求，加快旅游项目建设，加强旅游市场营销，争取天台山、神仙居成为5A级景区，支持临海台州府城文化旅游区、温岭长屿硐天景区创建5A级景区，加快市绿心、大陈岛旅游度假区、石塘半岛旅游区、三门中国亚热带植物园建设，打造仙居绿道休闲产业经济带，推动大陈全岛旅游化，争取成为国家海峡两岸交流基地。引导银行业金融机构加快金融产品创新和还贷方式创新，支持地方城市商业银行做专做强，规范发展小额贷款公司和农村资金互助社，推进农合机构股份制改革，大力发展农村普惠金融。加强商贸基础设施建设，整合完善商贸流通体系，推动专业市场转型发展，加快农村商业网络建设，繁荣商贸经济，社会消费品零售总额增长11%以上。市中心粮库一期工程竣工投用。加快现代物流发展，推动物流企业规模化、集约化，促进快递物流园区集聚，做大做强骨干快递企业。推进15个省市服务业集聚区建设。推动会展业发展。培育总部（楼宇）经济。保持房地产业平稳健康发展。

　　（三）实施融入国家开放战略行动。

　　扩大开放合作交流。主动融入“一带一路”和长江经济带战略布局，争取一批项目纳入国家战略规划。加强与上海自贸区对接，承接长三角通关一体化。落实外经贸促进政策，出口增长6%以上。深化口岸“大通关”建设，完善出口基地公共服务平台，推行进出口诚信企业联合培育推荐工作模式，加强出口信用保险和信保融资应用，拓展外经贸活动平台。加强企业境外营销网络建设，稳定传统市场，拓展新兴市场。优化出口结构，支持自有品牌、自主知识产权和高新技术产品出口。加快浙江中非国际经贸港等综合服务平台建设。争取成为大陆居民赴台湾自由行城市。改进招商引资方式，整合招商资源，强化精准招商、服务招商。加强与世界500强、央企、大型民企的战略合作，积极引进一批产业大项目。发挥异地商会优势，深入实施“浙商回归”，引进省外产业项目到位资金210亿元，着力打造台州商人总部中心。千方百计留住本地优质企业，支持做大做强。

　　加快港口开发开放。完成《台州港总体规划》修订的报批，调整海门港区功能布局，推进台州港一体化。加快头门港区开发，动工建设头门作业区起步码头二期、围填海工程，做好北洋涂二期围垦工程、头门岛南北侧成陆工程，推动口岸开放和综合保税区申报工作。加快228国道泽国至大麦屿疏港公路建设，建成大麦屿港区对台湾海上直航联检大厅、海峡两岸商品交易物流中心，推动两岸车渡直航试点。完善港口集疏运体系。创新港口运营机制。启动椒江口疏浚围垦开发前期。

　　建设海洋经济强市。引进培育海洋新兴产业，择优发展临港重化工业，积极培育海运、港航服务业，推进航运集聚区建设，加快临港装备制造基地、能源建材港航物流基地、海洋清洁能源产业基地建设。推进玉环海岛统筹发展试验区和浙台经贸合作区建设，深化玉环国家海洋生态文明建设示范区建设。推动三门湾区域合作。

　　（四）实施美丽台州建设行动。

　　强化环境整治。实施消灭黑臭河和劣V类水三年行动计划，深入推进“五水共治”，健全河长制，巩固“清三河”成果，加快截污纳管、污水处理厂提标改造、农业面源污染防治。实施大气污染防治六大专项行动，深化重点行业和重点区域整治，巩固椒江岩头化工恶臭整治成果，实现江口告别恶臭，推进临海川南、天台坡塘、仙居城南等医化园区告别恶臭整治，消除人民群众“心肺之患”。加快工业固废集中处置中心建设。推动金属资源再生产业基地国家“城市矿产”示范园区建设，实现“圈区管理”。实施永久性基本农田土壤治理三年行动计划，创建国家土壤污染综合防治示范区。完善生态环境质量考核制度，严厉打击环境违法行为。

　　保护自然生态。完成环境功能区划编制，严格划定生态红线。健全森林、湿地资源开发保护管理机制，加强生态公益林建设，推进鉴洋湖、三江、漩门湾、始丰溪等国家湿地公园保护开发。实行最严格水资源管理制度，加强长潭水库等饮用水源保护，县级以上集中式饮用水水源地水质全部达标。加快台州湾、三门湾、乐清湾污染综合治理。推广生态财政奖惩制度，健全椒江流域生态补偿机制，开展永安溪、始丰溪、灵江治理和生态保护。深入开展“四边三化”行动，创建省生态市、国家森林城市。

　　促进节能减排。落实区域限批和“一票否决”制度，健全新型环境准入制度。加快节能减排重点项目建设，加强重点领域和公共机构节能，坚决淘汰落后产能，积极创建省循环经济示范城市（县）。实施燃煤电厂清洁排放技术改造三年行动计划，推进火（热）电脱硫脱硝达标治理。编制市县两级温室气体清单，实施重点单位温室气体排放报告制度。探索建立减碳工作评价考核机制，开展区域用能权交易、水权交易试点。推行阶梯式水价、气价制度，创建省节水型城市。

　　（五）实施全面深化改革行动。

　　激发市场主体活力。积极创建国家小微企业金融服务改革创新试验区，完善小微金融研究院运行机制，编制小微金融指数，争取设立民营银行，建立地方金融业发展资金池，推动法人金融机构信贷资产证券化，发挥小微企业信保基金和信用信息共享平台功能，支持设立民间融资服务中心，切实解决小微企业融资贵、担保难问题。构建民间投资和创业便利化、贸易自由化发展新机制，争创国家民间投资改革创新示范区。深化商事登记制度改革，推动“大众创业、万众创新”。推进公共资源市场化改革。全面开展资源要素市场化配置改革，推广要素分层定价制度。深化国资国企改革，全面推行市属企业绩效考核。

　　深化社会领域和农村改革。加快户籍管理制度改革，有序推动农业转移人口市民化。大力推进社会资本兴办医疗、教育、文化、体育、养老，创新文化产业发展机制。完善市区警务一体化改革，推进水务、教育体制改革。深化农村产权制度改革，推广“三权到人（户）、权跟人（户）走”制度，力争乡镇一级产权交易平台全覆盖，推进农村宅基地确权登记发证，加快村经济合作社股份合作制改革，完善农村宅基地有偿使用、有偿退出机制，探索跨村跨镇置换机制，赋予农民更多财产权利。加强土地流转市场建设，引导土地经营权规范有序流转。抓好温岭市国家农村土地承包经营权确权登记试点。扶持发展农村集体经济。完善现代林业发展机制。

　　推动政府职能转变。深化“四张清单一张网”改革，横向拓展，纵向延伸，建立健全动态调整机制，拓展提升政务服务网功能。深化行政审批制度改革，健全以市场准入标准为基础，以完工验收、开工许可为主要内容的事中事后监管制度。合并组建市卫生和计生委。开展不动产统一登记职责整合、机构建设。探索跨部门综合执法机制。完善市区发展协调机制，建立联席会议制度。改革预算管理制度，完善预算公开和绩效管理制度，加强地方政府性债务管理，推进财政专项资金竞争性分配、财政性资金存放和社保基金保值增值管理改革。健全政府向社会购买服务机制。

　　（六）实施平台构建项目攻坚行动。

　　加速集聚区大平台构建。紧盯“五年出形象”目标，加快一期吹填区块路网、河网、管网、电网等设施建设，提高产业承接能力。加大高端装备制造、新材料、休闲旅游等产业招商力度。加快中小企业创业园、小微企业科创园建设，实现首批企业投产。启动南洋新材料产业园建设。创建省高新技术产业园区。启用集聚区行政服务中心，建成商业服务中心。加快“一湖三园”建设，推进月湖绿岛二期吹填工程，启动台州湾湿地公园、现代农业观光园、体育公园建设，促进产城融合发展。优化开发区（工业园区）发展规划、产业定位，鼓励兴建“一区多园”，促进产业集群、企业集聚。

　　加快重大项目建设。加强重大项目前期，深入实施省“411”扩大有效投资行动计划，固定资产投资增长13%以上。吉利豪情汽车迁建项目建成投产，加快吉利V汽车、广汽吉奥项目建设，动工建设北车台州配套产业园，力争东海油气登陆项目落户，继续努力推进炼化一体化项目前期。争取三门核电1号机组并网发电，确保台州第二电厂一期并网发电。推进甬台温天然气（成品油）管道一期建设。栅岭汪排涝调蓄工程河道基本连通、隧道全面贯通。加快洪家场浦排涝调蓄工程建设，推进方溪水库、盂溪水库、大田平原排涝、东官河综合整治工程，争取朱溪水库动工，完成东屏水库可研报批。

　　加强资源要素保障。组建国有资本运营公司，实现国资监管全覆盖。建立产业基金、城市发展基金、铁路建设基金。创新融资方式，推广PPP模式，激活社会投资。对接多层次资本市场，大力发展债券和股权融资，拓展企业融资渠道，力争直接融资120亿元。优化信贷结构，新增贷款550亿元。加快城镇低效用地和“三改一拆”拆后土地再利用，加大批而未供、供而未用土地处置力度，盘活存量土地5000亩。推进“812”土地整治工程，造田造地新增耕地1.8万亩。

　　（七）实施社会事业进步行动。

　　加快发展社会事业。推进“教育现代化市”创建，争创2个省教育基本现代化县。实施学前教育第二个三年行动计划，推进省义务教育标准化学校建设。坚持普通高中优质特色发展，适应新高考制度，深化教育教学改革。大力发展职业教育，推进产教融合、校企合作，启动台州现代职业教育创业园区建设。推动高等教育发展。探索民办学校分类管理，增加优质教育资源，深入实施百万学生素质提升工程。加快台州市直高中和开发区、集聚区新学校建设，启动台州科技职业学院二期工程建设，建成台州电大新校园，实现台州学院椒江校区校园整体合围。设立台州学院直属附属医院。推进卫生强市建设，深化公立医院综合改革，加快实施医疗资源“三千提增”计划，加强基层卫生工作，积极实施分级诊疗制度，鼓励医师多点执业。加强重点传染病防控和重点慢性病规范化管理，提高全民健康素养。推动中医药发展。加快市公共卫生中心、市妇女儿童医院（市妇幼保健院）建设，推进市立医院、市第一人民医院、台州医院等新院区建设，市第二人民医院投用。巩固出生人口性别比治理成果，人口自然增长率控制在6％。以内。推进公共文化服务标准化、均等化建设，完善公共文化基础设施。推进新闻出版事业发展，支持传统媒体与新媒体融合。鼓励和扶持文化精品创作，开展海洋文化研究，促进地方特色文化发展，加强文化遗产保护，增强文化认同，扩大区域文化影响。提升文化发展指数。启动台州大剧院项目前期，推进文化创意产业园建设，建成椒江文化创意园。推进体育强市建设，广泛开展全民健身运动，加快体育产业发展，力争实现体育强县全覆盖。进一步夯实统计基础。加强民族宗教、外事侨务、对台事务工作，做好档案、地方志和气象工作。

　　完善就业和社保体系。健全创业创新服务支持体系，实施大学生创业引领计划，做好高校毕业生、困难群众等重点群体就业工作，扶持大学生创业实体300家以上，实现零就业家庭动态清零，城镇登记失业率控制在4%以内。推进社保扩面，实施城乡居民一体的基本医疗保险制度，全面落实城乡居民大病保险制度，实现城乡各类基本养老保险制度依规有效衔接，稳妥跟进机关事业单位养老保险改革。健全以城乡居民最低生活保障制度为基础的社会救助体系，消除救助“盲区”、兜住保障“底线”。加快社会养老服务体系建设，推动医养融合发展。支持残疾人事业发展，加快残疾人托养中心建设。推进棚户区改造，加快保障性安居工程建设，确保公平分配，强化后续管理，扩大住房公积金制度覆盖面，构建多层次住房保障体系。完善最低工资保障机制，健全企业工资集体协商机制。保障妇女儿童权益，加强未成年人保护。倡导慈孝文化，发展慈善事业，继续开展“慈善公益日”活动。做好对口支援和对口帮扶。

　　推进社会治理创新。坚持系统治理、依法治理、综合治理、源头治理，促进法治、德治、自治融合。深化“网格化管理、组团式服务”，推进“平安台州”建设。深化“三严三治三强”工作，推进基础信息化、警务实战化、执法规范化和队伍正规化建设，完善全程式清障护航机制，健全立体化治安防控体系。推广相对集中行政处罚权工作，探索基层综合执法。健全社会矛盾纠纷预防化解机制，完善调解、仲裁等多元化纠纷解决机制，加强行业性、专业性人民调解组织建设。加强政府热线运行管理。推进信访改革，提高网上投诉办理质量，完善涉法涉诉信访依法终结制度，切实化解信访积案。防范企业“资金链”、“担保链”断裂风险，严厉打击恶意逃废债行为。创新和完善流动人口管理服务。深入实施“六五”普法。创建省社区矫正规范化示范区。推行村（社区）工作准入和社区居民代表会议制度，健全基层民主协商机制，推动社会组织健康有序发展。制定食品药品“智慧监管”系统三年建设规划，深化基层责任网建设，严厉打击制售假劣食品违法犯罪行为。强化烟花爆竹销售燃放管理。落实企业主体责任，深化隐患排查治理，严防重大安全生产事故，确保安全生产三项指标持续下降。加强防灾减灾能力，建成市防汛应急（森林消防）指挥中心，提升突发事件应急管理水平。大力支持驻台部队建设，做好国防教育、国防动员、民兵预备役和人防、海防工作。推进军民融合深度发展，深化双拥工作，努力蝉联全国双拥模范城称号。

　　（八）实施新农村建设和农民持续增收行动。

　　加快农业现代化。稳定粮食播种面积，保障主要农产品供给。开展基本农田保护补偿机制试点，划定永久基本农田。抓好农业“两区”建设，完善农业水利基础设施，新建高标准基本农田9.54万亩、粮食生产功能区9.6万亩、省现代农业综合区2个。推动农业全产业链建设，大力发展现代种业，推进农业特色产业“三化”示范村建设。深入实施“肥药双控”工程。扶持发展农业龙头企业。开展示范性家庭农场创建，推进股份合作农场试点，提升农民专业合作社发展，创建一批新型畜牧业合作组织。加强基层农业公共服务中心建设，加快构建“三位一体”农合组织体系。推进农业保险扩面。推进与省农科院科技合作三年行动计划。开展创新农作制度示范乡镇建设。鼓励大学生到农村创业。发展生态渔业、设施渔业，推动现代水产加工业发展，加快标准渔港和渔港经济区建设。深化渔场修复振兴暨“一打三整治”行动，全面完成涉渔“三无”船舶取缔任务。

　　推进美丽乡村建设。深化“清洁家园”活动，扩大农村垃圾减量化、资源化处理试点，完成1691个村生活污水治理。连线连片推进美丽乡村建设，新建成20个美丽乡村精品村。加快农房改造、“空壳村”、“空心村”整治和“坡地村庄”建设，开展20个省美丽宜居示范村创建，推进天台全国休闲农业与乡村旅游示范县创建。加强历史文化村落保护利用，打造乡村文化品牌。完善农村基础设施，推进农村社区公共服务体系建设。

　　拓宽农民增收渠道。切实增加农民家庭经营性、工资性、转移性和财产性收入。推广“吃住农家乐、游玩大景区”模式，大力发展民宿型农家乐特色村。推动农村电子商务发展，促进农产品流通销售。深入实施“农民培训”工程，培训农民5万人。实施低收入农户收入倍增计划，开展“十有六保”精准扶贫，启动建设32个扶贫示范村，完成扶贫搬迁1万人。推进水库移民创业致富奔小康行动计划，打造年产值百万以上移民产业基地10个。

　　（九）实施为民办实事行动。

　　一是“五水共治”。完成296.2公里黑河、臭河治理，并巩固提高；加强截污纳管，污水处理率提高4个百分点，劣V类水质断面比例下降1.5个百分点；加快推进农村生活污水治理，新增农村生活污水治理受益农户18.5万户。二是雾霾治理。淘汰3.3万辆黄标车，实现基本淘汰目标；全面淘汰改造燃煤小锅炉；确保PM2.5浓度下降5个百分点以上。三是食品安全。新改造25家城镇农贸市场为放心市场；47家城区农贸市场建成食品安全快速检测室，免费向公众开放；大型、特大型餐饮企业和学校食堂的阳光厨房建设率达到30%；加强食品安全基层责任网络建设，配强乡镇（街道）、村（社区）两级食品安全管理队伍。四是社会保障。打好扶贫攻坚战，基本消除家庭人均年收入低于4600元的贫困现象；城乡居民最低生活保障月标准分别增长8%、10%；将无人抚养和贫困家庭重残、重病儿童优先纳入儿童福利保障范围；将就业年龄段无固定收入残疾人生活补贴月标准提高到125元。五是现代养老。新增机构养老床位2200张，建设护理型床位2400张，新建500个城乡社区居家养老服务照料中心。六是教育卫生。标准化中小学校比例提高到80%；在280所中小学新建加热保温饮水设施；改造完成30所中小学校塑胶跑道运动场地；实施全科医生签约服务制度。七是公共交通。加强农村公路提升改造，提升技术等级200公里，完成路面维修300公里，实施安保工程300公里，建成港湾式停靠站300个；推进市区公交发展，加密公交车次，建设公交换乘场站7个、港湾式停靠站24个，市区公交分担率提高2个百分点以上。八是文化体育。新增农村文化礼堂136个；公共体育设施和学校体育场地设施免费或低收费向社会开放。九是公益救助。各县市区财政设立临时救助专项资金。十是便民服务。在城乡社区新建850个“E邮柜”等电子商务投递终端；加快推进城市垃圾分类处理，市区垃圾分类收集覆盖面达到50%；农村应急广播体系新增2181个行政村，实现全覆盖；推进公共法律服务体系建设。

　　（十）实施法治政府和信用台州建设行动。

　　强化依法行政。严格执行市人大及其常委会决议决定，自觉接受人大法律监督、工作监督和政协民主监督，认真办理人大代表建议、政协提案。拓宽民主党派、人民团体和社会各界人士建言献策的渠道，主动接受司法监督和公众监督。健全依法决策机制，把公众参与、专家论证、风险评估、合法性审查、集体讨论决定确定为重大行政决策的法定程序，确保政府决策遵循法治轨道、符合法治要求。严格行政执法程序，规范行政执法裁量权，健全行政复议机制，加大行政执法过错责任追究，做到法无授权不可为、法定职责必须为。

　　推进科学施政。完善政府科学决策程序，统筹政府智库建设，注重发挥决策咨询等专家作用，建立政府法律顾问制度，对于涉及公众利益的重大决策实行事前论证、事中监督、事后评估，加强绩效管理，引入第三方评估，健全重大决策责任追究机制和倒查机制，提升科学施政、民主决策水平。强化行政权力的制约监督，加强审计监督，运用“制度+科技”办法，完善行政权力制约监督体系。推进决策公开、执行公开、管理公开、服务公开、结果公开，确保行政权力在阳光下运行。完善工作执行体系，全面开展“对标行动”，重实干、敢担当、创一流。

　　严格廉洁从政。深入推进党风廉政建设和反腐败斗争，严明纪律，严惩腐败。认真执行党风廉政建设责任制，切实履行“一岗双责”。全面落实民主决策、重点领域监管、防止利益冲突、农村党风廉政建设等制度，加强廉政风险防控，把权力关进制度的笼子里。践行“三严三实”，巩固和扩大党的群众路线教育实践活动成果，持之以恒执行中央“八项规定”和纠正“四风”，切实抓好中央和省委巡视组反馈意见的整改落实工作，健全作风建设常态化制度。深入开展正风肃纪专项行动，坚决纠正损害群众利益的不正之风，严肃查处违法违纪案件。

　　打造信用台州。加强道德文化宣传教育，倡导社会主义核心价值观。加强信用体系建设，大力推进政务诚信、商务诚信、社会诚信和司法公信建设，建立健全覆盖全社会的征信体系，推动信用信息系统建设及互联互通。深化国家知识产权示范城市建设，加强知识产权执法和监督。建立守信激励和失信惩戒机制，褒扬诚信行为，建立失信“黑名单”制度，加大失信惩戒力度，提高失信成本，在全社会形成守信光荣、失信可耻的良好氛围。

　　各位代表，今年是“十三五”规划谋划之年。我们要深入调研，集思广益，认真研究一批对经济发展和结构调整全局带动性强的重大工程，对推进社会建设、生态环保、民生改善作用显著的重大项目，对解决突出矛盾、增进公平效率有力有效的重大政策，认真编制“十三五”规划，绘就现代化港湾都市区的宏伟蓝图。

　　各位代表，新起点肩负新重任，新常态要有新状态。让我们紧密团结在以习近平同志为核心的党中央周围，在中共台州市委的坚强领导下，高举改革大旗，弘扬法治精神，凝聚赶超力量，为建设现代化港湾都市区而努力奋斗！
